# The Use of AlphaFold for *In Silico* Exploration of Drug Targets in the Parasite *Trypanosoma cruzi*

**DOI:** 10.3389/fcimb.2022.944748

**Published:** 2022-07-14

**Authors:** Albert Ros-Lucas, Nieves Martinez-Peinado, Jaume Bastida, Joaquim Gascón, Julio Alonso-Padilla

**Affiliations:** ^1^ Barcelona Institute for Global Health (ISGlobal), Hospital Clinic - University of Barcelona, Barcelona, Spain; ^2^ Departament de Biologia, Sanitat i Medi Ambient, Facultat de Farmàcia i Ciències de l´Alimentació, Universitat de Barcelona, Barcelona, Spain; ^3^ CIBERINFEC, ISCIII—CIBER de Enfermedades Infecciosas, Instituto de Salud Carlos III, Madrid, Spain

**Keywords:** chagas disease, *trypanosoma cruzi*, drug discovery, AlphaFold, target deconvolution

## Abstract

Chagas disease is a devastating neglected disease caused by the parasite *Trypanosoma cruzi*, which affects millions of people worldwide. The two anti-parasitic drugs available, nifurtimox and benznidazole, have a good efficacy against the acute stage of the infection. But this is short, usually asymptomatic and often goes undiagnosed. Access to treatment is mostly achieved during the chronic stage, when the cardiac and/or digestive life-threatening symptoms manifest. Then, the efficacy of both drugs is diminished, and their long administration regimens involve frequently associated adverse effects that compromise treatment compliance. Therefore, the discovery of safer and more effective drugs is an urgent need. Despite its advantages over lately used phenotypic screening, target-based identification of new anti-parasitic molecules has been hampered by incomplete annotation and lack of structures of the parasite protein space. Presently, the AlphaFold Protein Structure Database is home to 19,036 protein models from *T. cruzi*, which could hold the key to not only describe new therapeutic approaches, but also shed light on molecular mechanisms of action for known compounds. In this proof-of-concept study, we screened the AlphaFold *T. cruzi* set of predicted protein models to find prospective targets for a pre-selected list of compounds with known anti-trypanosomal activity using docking-based inverse virtual screening. The best receptors (targets) for the most promising ligands were analyzed in detail to address molecular interactions and potential drugs’ mode of action. The results provide insight into the mechanisms of action of the compounds and their targets, and pave the way for new strategies to finding novel compounds or optimize already existing ones.

## Introduction

Chagas disease, caused by the parasite *Trypanosoma cruzi*, is a potentially life-threatening disease with several socioeconomic, environmental and public health issues (World Health Organization, 2022). It is endemic in Latin America where it exerts its highest burden. Moreover, owing to migration in recent decades, it has spread to other non-endemic regions becoming a global health issue. Approximately 6-7 million people worldwide are infected with *T. cruzi*, and 10,000 people die annually from such infection (World Health Organization, 2022). Its acquisition occurs by vector, congenital, iatrogenic or oral routes (World Health Organization, 2022). Once infected, individuals go through a short (4-8 weeks) acute phase that is characterized for the appearance of non-specific mild symptoms or an absence of symptomatology which makes it go undiagnosed. Then, the disease progresses to a chronic phase which can be silent for life or, in 30-40% of the patients, manifest with cardiac and/or digestive alterations that can lead to the formation of mega-syndromes and death if untreated (Prata, 2001).

For the last 50 years, the nitroheterocyclic drugs benznidazole (BNZ) and nifurtimox (NFX) have been the only drugs available to treat Chagas disease. BNZ and NFX are prodrugs that act through the formation of free radicals and electrophilic metabolites generated when its nitro group is reduced to an amino group by the action of nitro-reductases (Wilkinson et al., 2008). Both drugs have shown to be effective when administered to early infections and are well tolerated by infants (Prata, 2001). However, their efficacy diminishes at the chronic stage and the appearance of toxic side effects usually leads to treatment interruption (Alonso-Padilla et al., 2019). Thus, there is an urgent need for new drugs for Chagas disease.

Drug development is a long and expensive process handicapped by high attrition rates. In the drug discovery cascade, compounds are first evaluated through *in vitro* assays prior its evaluation at preclinical and clinical trials. At this early stage, two strategies are typically undertaken to identify hit compounds: target-based or whole cell phenotypic assays (Martínez-Peinado et al., 2020). The latest are usually preferred over target-based approaches as those represent a more holistic insight with higher translational rate to *in vivo* efficacy assessment (Martínez-Peinado et al., 2020). However, phenotypic approaches usually require additional steps to identify the molecular target, not just for elucidating the mechanism of action, but also to aid in the rational design of the drug and allow efficient structure-activity relationship (SAR) studies (Terstappen et al., 2007). The process to identify the molecular target, termed target deconvolution, may entail expression cloning-based methods, protein microarrays, RNAi/CRISPR screening or radioactive compound-binding assays, among others (Kubota et al., 2019). However, these experiments are time- and resource-extensive, and computational alternatives commonly known as *in silico* target prediction or molecular docking studies have gained considerable attention in last years (Kubota et al., 2019). This is particularly the case in Neglected Tropical Diseases (NTDs) drug discovery research, like that for Chagas disease, where developmental costs must be kept necessarily low due to the scarce funds available. Notably, these computational strategies have been strengthened thanks to the increasing availability of pathogen sequences and genome-scale functional datasets (Crowther et al., 2010).

Chagas disease, as other NTDs, suffers from a lack of well-characterized and validated targets that has hampered drug development (Chatelain and Ioset, 2018). Among *T. cruzi* identified targets there are the following enzymes: triosephosphate isomerase (TIM), sterol 14α-demethylase (CYP51), dihydroorotate dehydrogenase (DHODH), cruzipain, trypanothione reductase (TR), superoxide dismutase (Fe-SOD), pteridine reductase (PTR) and dihydrofolate reductasethymidylate synthase (DHFR-TS) (Beltran-Hortelano et al., 2022). Interestingly, the recent failure of posaconazole, inhibitor of *T. cruzi* CYP51, in clinical trials has highlighted the challenge of molecular target validation for Chagas drug development; and such target could ultimately be validated if associated with a curative profile (Chatelain and Ioset, 2018).

AlphaFold is a recently developed software for the prediction of protein 3D structures from their genetic sequence (Jumper et al., 2021). The AlphaFold Protein Structure Database has the entire human proteome, as well as the entire proteomes of other 20 widely studied organisms such as *Escherichia coli, Trypanosoma brucei* or *T. cruzi*. Specifically, it is home to 19,036 protein models from *T. cruzi* (Varadi et al., 2022). Thus, it has emerged as a very valuable tool to predict potential targets and hypothesize mechanisms of action of known compounds. In this proof-of-concept work, we have used docking-based inverse virtual screening with AlphaFold *T. cruzi* protein models to find prospective targets for a pre-selected list of compounds with known anti-trypanosomal activity in clinical trials or in chronic *in vivo* models of *T. cruzi* infection. The goal is to assess the usefulness of AlphaFold models for *in silico* drug discovery pipelines, as well as computationally validating the targets described for this list of compounds.

## Methods

### Generation of the *T. cruzi* library of potential targets

First, a list of genes of interest was created from the TryTrip database (Aslett et al., 2010; Amos et al., 2022). All genes from *T. cruzi* CL Brener Esmeraldo-like strain, member of the pathogenic Discrete Typing Unit II (DTU II), were searched. We further selected genes which expression was above the 10% percentile in any of the samples of the three experiments where trypomastigote or amastigote samples were available (Smircich et al., 2015; Li et al., 2016; Belew et al., 2017). Additionally, genes were also selected if at least one peptide was detected in any of the two mass spectrometry proteomic experiments for trypomastigotes and/or amastigotes (Atwood et al., 2005; Marchini et al., 2011). Genes without a UniProtKB ID were discarded, since the AlphaFold Protein Structure Database only contains models with an entry in UniProtKB (The UniProt Consortium, 2021).

All protein models in PDB format for the *T. cruzi* CL Brener proteome were downloaded from the AlphaFold Protein Structure Database downloads section on October 20^th^ 2021 as a compressed tar file. Models with a UniProtKB ID that did not match any of the selected genes were discarded. Selected genes without a model were a consequence of their products’ length being larger than 2,700 residues, and since AlphaFold models were not available for such lengths, these were discarded too. Conversion to the pdbqt format used for docking was done with Open Babel (O’Boyle et al., 2011), adding hydrogens with a pH of 7.4 and using the Gasteiger method to add partial charges. Binding pocket prediction was performed using P2Rank (Krivák and Hoksza, 2018) with standard settings. Pockets with a probability score (as given by P2Rank) above 0.1 were considered as candidates for binding sites. For each model, the pocket with the highest probability score was selected as the binding site. Structures without predicted pockets, or predicted pockets with a probability score below 0.1, were discarded.

In order to assess the global model quality, the predicted Local Distance Difference Test (pLDDT) (Mariani et al., 2013) score of each α-carbon was extracted from the PDB files, and the proportion of residues with a pLDDT score above 70 (described as the threshold for good backbone prediction) (Tunyasuvunakool et al., 2021) was calculated. Only models with at least half of its total residues with a pLDDT score above 70 were considered for docking. Additionally, to assess the local quality of the binding pocket, the residues predicted to be part of the pocket by P2Rank were considered. Only models in which at least half of the residues in the pocket had a pLDDT score above 90 were kept. This stricter threshold is given by the fact that residues with a pLDDT score above 90 can be interpreted as having very high quality and correct side-chain orientation (Tunyasuvunakool et al., 2021). Finally, the Predicted Aligned Error (PAE) of the pocket residues was also analyzed. To do so, the mean PAE of each residue of the pocket (as specified by P2Rank) with the rest of the residues of the pocket was calculated, and the overall mean PAE was obtained. Any model with a mean pocket PAE above 5 Å was discarded.

### Identification of the list of ligands

In order to select suitable ligands, we undertook a search of publications in PubMed/MEDLINE and ClinicalTrials.gov databases. Searches in ClinicalTrials.gov were performed in January 2022 under the search term [Chagas disease]. We selected only those drugs in clinical trials with previously reported anti-*T. cruzi* activity (**Table S1**). Searches in PubMed/MEDLINE were performed from September to November 2021 and were restricted to publications published between 2015 and 2021. The search terms used were [*Trypanosoma cruzi*] AND [Drug] OR [Compound] OR [Natural product]. We performed a manual revision to select those that included anti-*T. cruzi* activity *in vivo* and prioritized those compounds that had inhibited the parasite equally or superior to the reference drug BNZ in a chronic model of the infection (**Table S2**). For those that were the result of chemical synthesis, we maintained the number of the compound reported in each publication and added a number from one to six to avoid name repetitions and to differentiate them (**Table S2**). Ligand structures were downloaded from PubChem (Kim et al., 2021) as 3D SDF files where possible, otherwise they were downloaded either as 2D SDF files or drawn using Avogadro (Hanwell et al., 2012). For the latter two, the final 3D conformation was obtained by using Avogadro’s Auto Optimization tool, using the UFF force field with 4 steps per update and the Steepest Descent algorithm until the energy differential (dE) fell below 0.001 for several seconds. Conversion to pdbqt format was also done using Open Babel, assigning charges using the Gasteiger method. For compounds C8-3 and C26-6, this method proved to be unsuccessful due to having selenium atoms, for which the EEM method was used instead. The size of the binding box for each ligand was optimized according to Feinstein and Brylinski (Feinstein and Brylinski, 2015), using a radius of gyration to box side ratio of 0.35, and rounding up to the nearest integer. The radius of gyration for each ligand was calculated using the Python RDKit (Landrum et al., 2021) library Descriptors3D module.

### Docking of targets to their described ligands

Docking simulations were performed using AutoDock Vina 1.1.2 (Trott and Olson, 2009). The exhaustiveness parameter was set to 8, and the energy range to 2. The search box center was chosen from the P2Rank predictions, and its size was calculated for each ligand as described above. For each receptor and ligand pair, ten docking repetitions were done with different random seeds. The best mode for each pair was chosen from the lowest docking energy of all the repetitions. This resulted in a matrix of *n* ligands by *m* receptors, with the best possible energy for each pair. To normalize results, a receptor-average *Z*-score matrix was calculated (Yang et al., 2009). For this, each value of the binding energy matrix was substituted by a *Z*-score using the formula:


$$Z_{ij}=\left(X_{ij}-{\bar{X}}_{i}\right)/SD_{i};$$

where *X_ij_* is the binding energy as given by AutoDock Vina for the receptor *i* and ligand *j* pair, ${\bar{X}}_{i}$ is the mean binding energy of receptor *i*, and *SD_i_* is the standard deviation for receptor *i*. Positive *X_ij_* values were ignored and considered as missing. The top 3% scored receptors in the *Z*-score combined matrix were chosen as the best putative receptors for each ligand. Results were visualized using PyMol (Schrödinger, 2015).

## Results and Discussion

**Figure 1:**
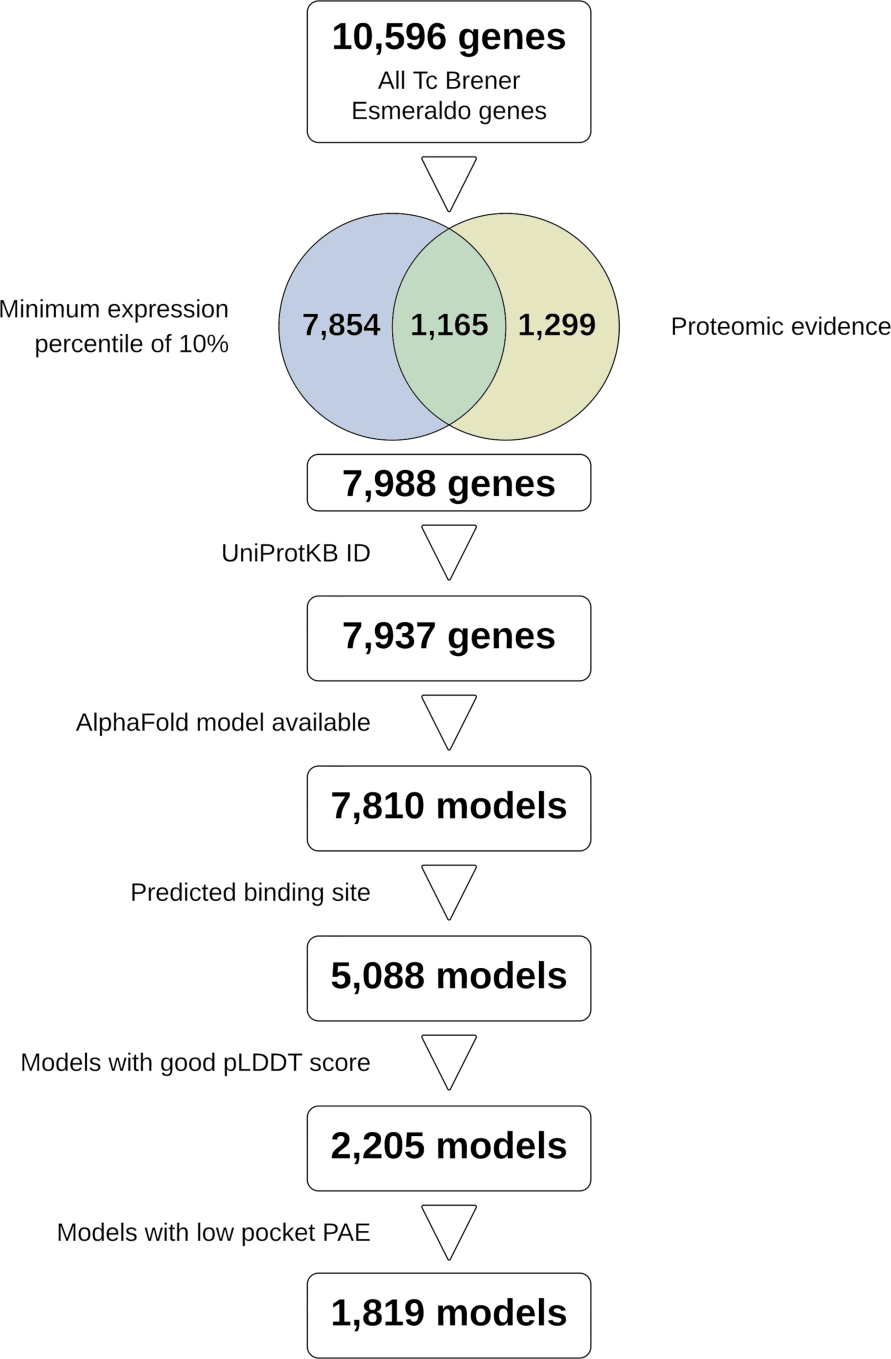
Study flowchart summarizing the steps followed to reach the models used in docking. Figure made in Lucidchart.

**Figure 2:**
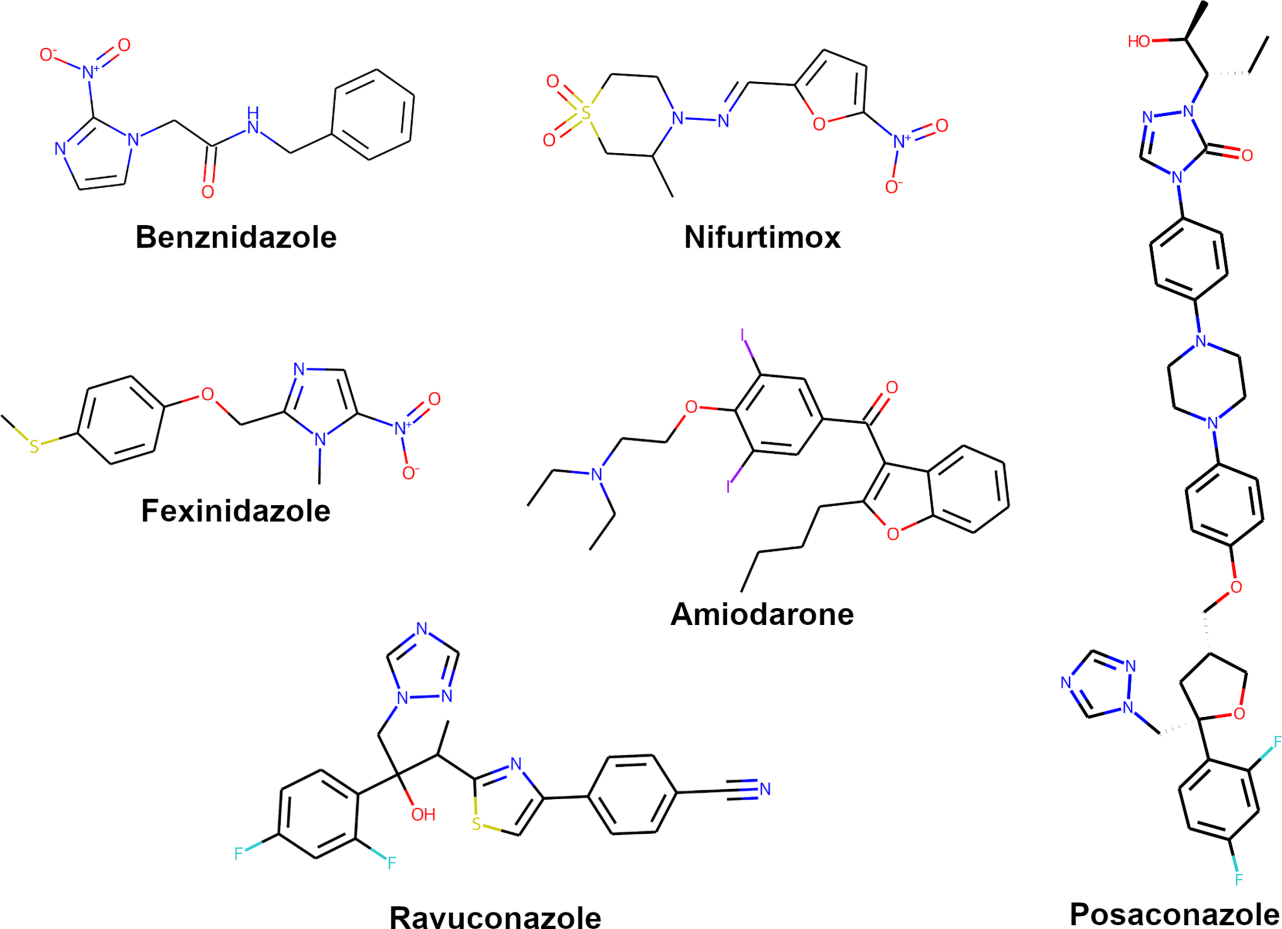
Structures of compounds with anti-*T.cruzi* activity used in clinical trials.

**Figure 3:**
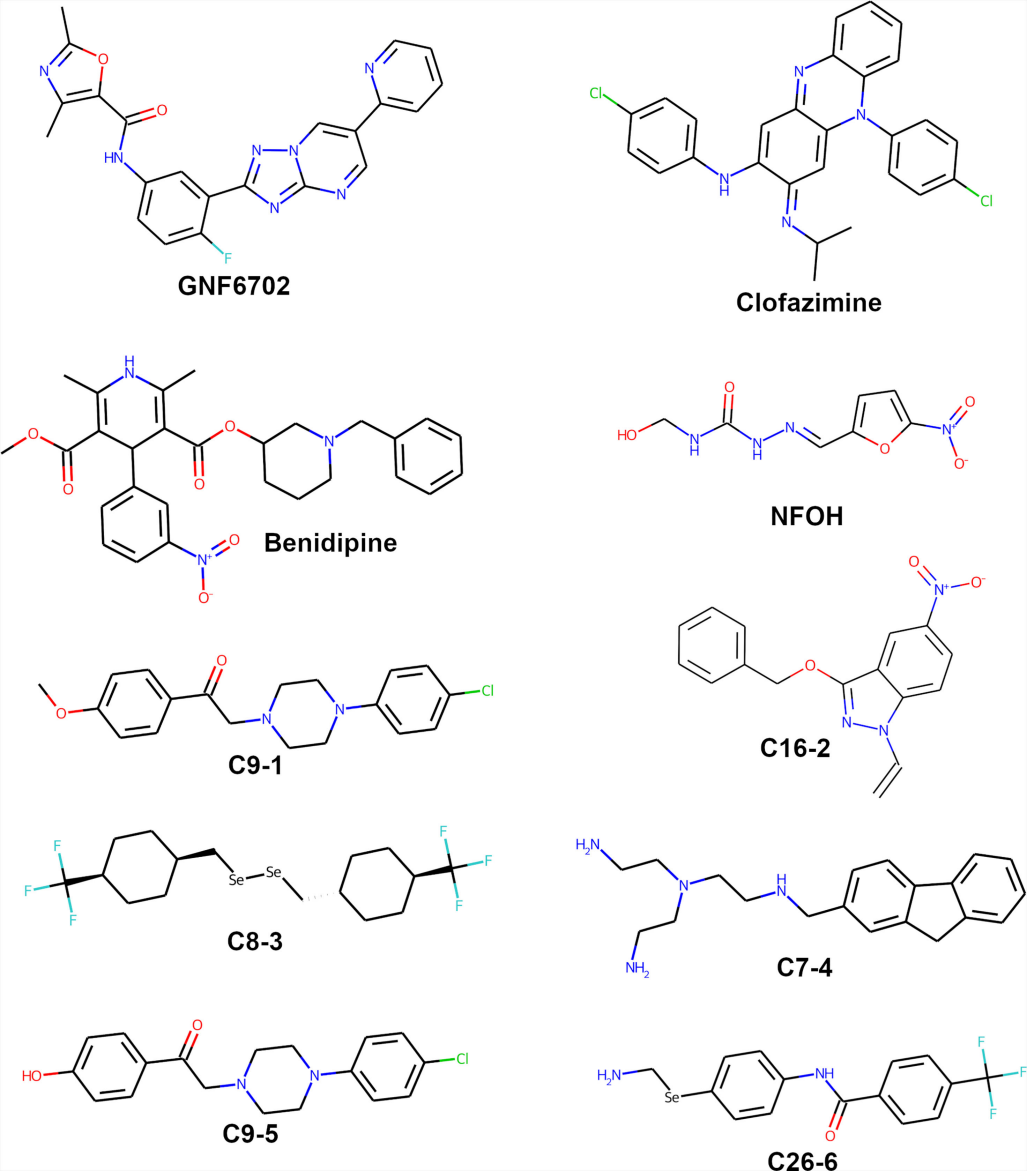
Structures of compounds with anti-*T.cruzi* activity used in chronic experimental models of the disease.

The final list of selected genes from TriTryp encompassed 7,988 entries filtered out of the 10,596 *T. cruzi* CL Brener Esmeraldo-like genes available. From these, 7,810 had a protein model available. A total of 5,088 models had a predicted binding site available, and after discarding models with low quality, given by low pLDDT scores or high PAE, only 1,819 models rested available for docking predictions (**Figure 1**). Our search and selection of ligands (as described in Methods) returned 16 compounds for the docking simulations (**Table 1**), 6 from clinical trials (**Figure 2**) and 10 from chronic models of infection (**Figure 3**). In total, 363,800 docking simulations were performed by AutoDock Vina, doing 10 repetitions for each receptor-ligand pair. Some ligands performed quicker than others, being the computation speed inversely proportional to their number of atoms and rotatable bonds. The lowest binding energy for each pair was selected as the best binding mode. Thus, the binding energy matrix generated contained 1,819 rows (receptors) and 16 columns (ligands) (**Table S3**). The average binding energy for each ligand, as well as their docking box edge size are shown in **Table 1**. The binding energy matrix was then normalized to a *Z*-score matrix using each receptor’s average binding energy and standard deviation (**Table S4**), and filtered to only keep the top 3% binders for each ligand as their putative receptors (**Table S5**).

**Table 1:** List of compounds used in this study.

Compound	Box size	Mean predicted binding energy (kcal/mol)	Described target
Benznidazole	10 Å	-7.07	Nitroreductase
Nifurtimox	12 Å	-6.91	Nitroreductase
Fexinidazole	13 Å	-6.50	Nitroreductase
Posaconazole	20 Å	-7.35	Ergosterol biosynthesis
Ravuconazole	14 Å	-7.59	Ergosterol biosynthesis
Amiodarone	13 Å	-6.39	Calcium homeostasis
GNF6702	16 Å	-8.87	Proteasome
NFOH	12 Å	-6.29	Nitroreductase
Clofazimine	15 Å	-7.51	Cruzipain
Benidipine	13 Å	-7.92	Cruzipain
Compound 9-1	16 Å	-7.33	FeSOD
Compound 16-2	11 Å	-7.05	Mitochondria
Compound 8-3	16 Å	-7.60	Mitochondria
Compound 7-4	15 Å	-7.00	Glycosome
Compound 9-5	14 Å	-7.33	FeSOD
Compound 26-6	13 Å	-7.04	Mitochondria

Taking a closer look at the compounds from clinical trials and good activity *in vivo*, and comparing their top binders with existing literature records, we found that some of them indeed have their experimentally validated receptors inside their corresponding top 3% putative receptors. In the following sub-sections we describe the results obtained with each class of inhibitors evaluated.

### Ergosterol biosynthesis inhibitors

Posaconazole is widely described to act upon the *T. cruzi* ergosterol biosynthesis pathway by inhibiting the CYP family enzyme lanosterol 14-alpha demethylase (TcCLB.510101.50, UniProtKB Q7Z1V1) (Lepesheva et al., 2011). In our analysis, we retrieved that same target in position 32 out of 54 in the top 3% receptors (targets) of posaconazole. While it would have been expected to find it in a higher position, its relatively low placement could be attributed to the fact that, as a CYP family member, the binding site of lanosterol 14-alpha demethylase contains a heme cofactor. This group directly interacts with posaconazole as seen in PDB structure 3K1O (Lepesheva et al., 2010). Even though AlphaFold models correctly predict the binding site of cofactors, they do not contain these molecules, which poses a certain limitation to their use in *in silico* docking. Advances to compensate for this have been made. For example, the yet unpublished AlphaFill (Hekkelman et al., 2021) can transfer cofactors from PDB into AlphaFold models based on sequence and structure similarity. In this regard, the AlphaFill model for the lanosterol 14-alpha demethylase correctly displays a heme group in the expected position, which would certainly improve the binding energy of posaconazole.

Ravuconazole is another compound that has been described to target the lanosterol 14-alpha demethylase (Lepesheva et al., 2011), but for this ligand we found that the expected target enzyme was outside the top 3% selected. Ravuconazole would be expected to also interact with the heme cofactor of the enzyme, and such low ranking could thus be reasonable. The fact we retrieved it at a much lower position in the list in comparison to posaconazole might be attributed to small inaccuracies in the binding pocket, which could probably be improved by allowing AutoDock Vina to use flexible pocket residues in its docking simulations. Unfortunately, such an approach was unfeasible in the current study due to computational time limitations.

### Compounds disrupting parasite calcium homeostasis

Amiodarone has been widely used to prevent arrhythmias in patients with Chagas cardiomyopathy (Stein et al., 2018). Nonetheless, it was recently described to have *in vitro* anti-parasitic activity and synergic activity with posaconazole in *in vivo* models of *T. cruzi* infection (Adesse et al., 2011; Benaim et al., 2021). Amiodarone has been described to act thought the disruption of intracellular calcium homeostasis, which has been identified as a potential therapeutic target in trypanosomatids (Benaim et al., 2021). More specifically, amiodarone collapses the mitochondrial electrochemical potential and prompts the alkalization of acidocalcisomes, increasing parasite intracellular calcium concentration (Benaim et al., 2021). In our analysis, we found the V-type proton ATPase subunit A TcCLB.509767.70 (Q4DSC7) at position 42 out of 54 in the top 3%. The V-type proton ATPase is involved in the acidification of the acidocalcisome by the uptake of H^+^ (Docampo and Moreno, 2011). Thus, this ATPase could be a possible target for this drug that would correlate with that described by Benaim et al. (Benaim et al., 2021). Additionally, we found a transporter (TcCLB.506369.20, UniProtKB Q4D047) located in the acidocalcisome membrane at position 36 out of 54. Amiodarone has also been reported to inhibit the oxidosqualene cyclase, a key enzyme in ergosterol biosynthesis. However, we did not find this enzyme in the top 3% of our analysis.

### Compounds activated by nitroreductase enzymes

**Figure 4:**
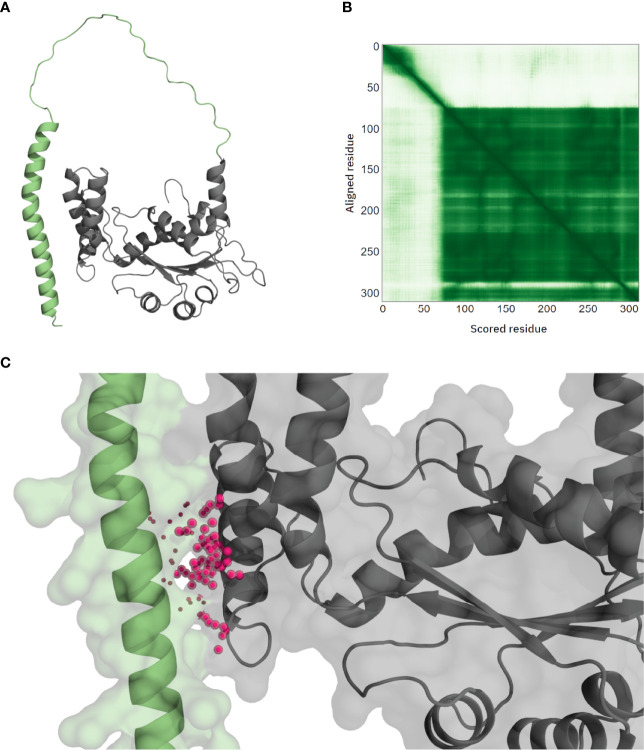
*T. cruzi* nitroreductase AlphaFold model. The relative position of the N-terminal domain (**A**, green residues) compared with the rest of the protein has very low confidence, as seen in the Predicted Aligned Error (PAE) plot **(B)**. The predicted pocket by P2Rank falls in the interaction between this domain and the rest of the protein (**C**, purple spheres), suggesting that this pocket might be an artifact.

Regarding BNZ, NFX, hydroxymethylnitrofurazone (NFOH) and fexinidazole, they are all described to be prodrugs, which upon being metabolized generate highly reactive intermediary compounds that can target many cellular components (Maya et al., 2007; Hall et al., 2011; Scarim et al., 2021). In particular, these drugs would be mainly metabolized by *T. cruzi* nitroreductase (TcCLB.510611.60, UniProtKB Q4D8D9) (Maya et al., 2007; Hall et al., 2011). However, the binding site detected by P2Rank in the AlphaFold model for this enzyme showed a high predicted aligned error (PAE), due to being formed between the N-terminal domain and the rest of the protein (**Figure 4**). This poor quality of the binding pocket prevented the model from being used in the screening, and thus it could not have been selected as a putative receptor for these ligands. Alternative enzymes that have been described to metabolize these drugs, such as dihydrolipoamide dehydrogenase, cytochrome P450 reductase, trypanothione reductase or prostaglandin F2α synthase (Hall et al., 2011), were neither selected for any of these ligands.

### Iron superoxide dismutase (FeSOD) inhibitors

Another ligand that has a suggested target in the top 3% putative receptors is the Manich base-type derivative C9-1, which is described to inhibit the iron superoxide dismutase enzyme (FeSOD) with an IC_50_ value of 6.5 µM (Martín-Escolano et al., 2018b). FeSOD is a trypanosomatid-exclusive enzyme that prevents oxidative stress caused by reactive oxygen species (ROS) and that considerably differs from its human homologue (Martín-Escolano et al., 2018b). Therefore, it has been considered a desirable druggable target. A TriTryp search for superoxide dismutase (SOD) enzymes for the CL Brener Esmeraldo-like strain resulted in six enzymes with matching annotation. However, only the TcCLB.511735.60 SOD (UniProt ID Q4CUQ5) was used for docking. Superoxide dismutase enzymes FeSOD TcCLB.509775.40 (Q4DCQ3), FeSOD TcCLB.511715.10 (Q4D5A6), FeSOD TcCLB.507039.10 (Q4CVN4) and SOD TcCLB.511545.120 (Q4DMR9), which pertain to the same ortholog group, were discarded because P2Rank failed to predict their binding pockets, while SOD TcCLB.511737.3 (Q4D5Z8) was discarded due to not having transcriptomic and/or proteomic evidence. However, all six showed high structural similarity upon alignment with PyMol (data not shown). A comparison with available PDB structures for the *T. cruzi* FeSOD 4H3E and 4DVH (both from TcCLB.509775.40) showed that this enzyme is in fact a homodimer, with the binding site located between its subunits. Unfortunately, the version of AlphaFold used in the AlphaFold Protein Database currently does not support multi-chain models. Thus, one of the limitations of our pipeline is the fact that protein complexes with binding sites situated in the interaction between chains will probably not have correct predictions. Despite this limitation, we found that TcCLB.511735.60 superoxide dismutase appeared in position 1 out of 55 of the best binders for C9-1, providing reliability to the methodology followed. Additionally, Manich base type-derivatives have been described as potent inhibitors of *T. cruzi* trypanothione reductase based on the ability of those compounds to interact with dithiol groups (Beltran-Hortelano et al., 2017). For C9-1, we found the trypanothione reductase (TcCLB.504507.5, Q4CMQ7) at the position 48 out of 55 in the top 3%, suggesting a multiple target for C9-1.

Upon looking at compound C9-5, which is also a Manich base-type derivative highly similar to C9-1 and also suggested to act on the FeSOD (Paucar et al., 2019), we were unable to find it in the top 3%. This might have been caused by the fact that, as described above, the FeSOD enzyme is found as a dimer, and the binding site used in the docking simulations did not reflect that reality. The only difference between these two ligands is an *O*-methyl group at one of the ends of the C9-1 molecule, where C9-5 only has a hydroxyl group. This gives C9-1 a bigger radius of gyration, and thus a larger docking box. It is possible that this allowed C9-1 to adopt a higher affinity mode in comparison to C9-5, hence the higher ranking of the FeSOD in that case.

### Cruzipain inhibitors

**Figure 5:**
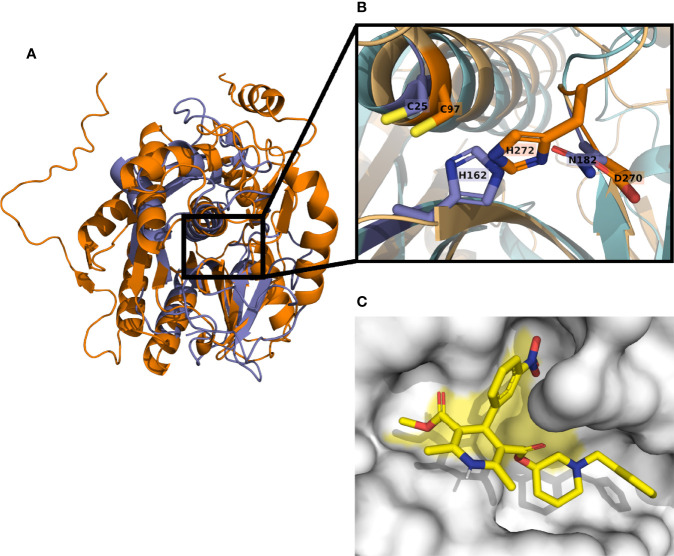
*T. cruzi* cysteine peptidase Q4D5K1 model aligned with cruzipain structure 4PI3. Structural similarities can be appreciated between cruzipain (blue) and the cysteine peptidase Q4D5K1 (orange) **(A)**. The catalytic triad in cruzipain is composed by residues C25-H162 -N182, and aligns with the putative catalytic triad C97-H272-D270 of the cysteine peptidase **(B)**. Docking predictions show benidipine (yellow) binding in the catalytic site (yellow area) of the cysteine peptidase **(C)**.

**Figure 6:**
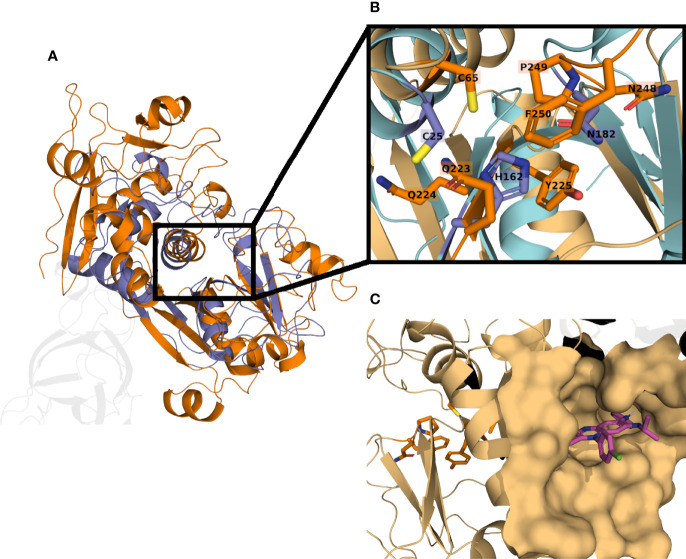
*T. cruzi* calpain-like peptidase Q4CMV9 model aligned with cruzipain structure 4PI3. Structural similarities can be appreciated between cruzipain (blue) and the calpain-like peptidase Q4CMV9 (orange) **(A)**; unaligned residues in the C-terminal domain are shown transparent. The calpain-like peptidase shows an inactive catalytic site, lacking the basic residue, typically a histidine, necessary to deprotonate the cysteine **(B)**. The binding site of clofazimine (magenta) is located far from the inactivated catalytic site (orange residues), suggesting another mechanism of action **(C)**.

Another two ligands considered were the cruzipain inhibitors clofazimine and benidipine (Sbaraglini et al., 2016). Cruzipain is a cysteine peptidase of *T. cruzi*, and three enzymes annotated as such were used in the docking simulations: major cysteine proteinase TcCLB.507603.270 (Q4DW02), cysteine peptidase TcCLB.506529.550 (Q4E5M4), and cysteine peptidase Tc CLB .507537.20 (Q4CV00). Cysteine peptidase TcCLB.507603.260 (Q4DW03) was discarded beforehand due to exhibiting a low mean pocket pLDDT. The docking analysis could not find any of the former three enzymes in the selected putative receptors for clofazimine and benidipine. Structural comparison of these enzymes with available cruzipain PDB structures 4PI3 and 3KKU showed that the binding site in the AlphaFold models is obstructed by residues between positions 80 and 110 approximately. UniProt annotation for cruzipain P25779 (corresponding to cysteine peptidase TcCLB.507603.260) indicates that residues 19 – 122 are in fact a propeptide, which would be cleaved in the mature protein. Thus, the cruzipain AlphaFold models we used do not reflect the reality of the protein, and the P2Rank pocket prediction could have not detected the binding site. Notwithstanding, it is interesting to find that in the top selected receptors for both ligands there are indeed other cysteine peptidases, in particular TcCLB.504107.10 calpain-like cysteine peptidase (Q4CMV9) in position 7 out of 52 for clofazimine; and TcCLB.509013.19 calpain-like cysteine peptidase (Q4CW01) and TcCLB.511527.50 cysteine peptidase (Q4D5K1) respectively in positions 32 and 38 out of 55 for benidipine. While the latter cysteine peptidase bears low sequence identity to cruzipains, its catalytic site hints to a structural similarity with them, as illustrated by the pairwise structure alignment using the TM-align algorithm of cruzipain PDB structure 4PI3 with the Q4D5K1 AlphaFold model (**Figure 5**). In particular, the catalytic triad appears to be roughly in the same positioning, Cys-His-Asn in the case of cruzipain, and Cys-His-Asp for the Q4D5K1 cysteine peptidase. It is then conceivable that benidipine would also show high affinity for this enzyme, being the binding site predicted by P2Rank located in the catalytic site. The calpain-like cysteine peptidases share some overall structural similarity to that of cruzipains; however, their catalytic site appears to be inactive, keeping the nucleophilic cysteine but without the amino acid dyad base needed to deprotonate it (**Figure 6**). Indeed, catalytically inactive calpain-like proteins are not uncommon (Dear et al., 1997; Ersfeld et al., 2005), and these might have another regulatory function in the parasite. Additionally, the binding site predicted by P2Rank for both calpain-like proteins is not located in the proximity of the catalytic site, suggesting that the inhibition by clofazimine and benidipine could be given by another mechanism.

### Proteasome inhibitors

**Figure 7:**
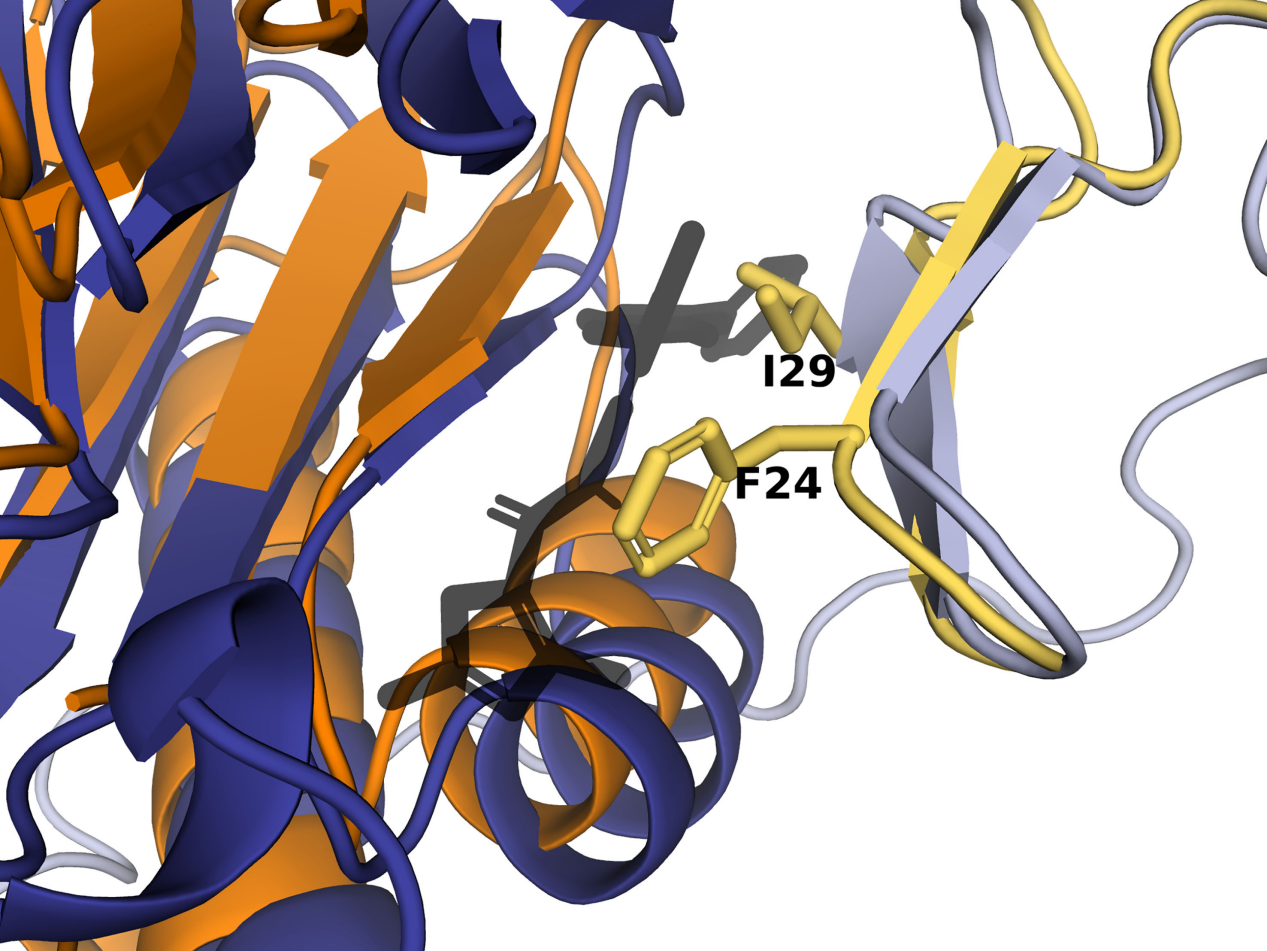
*T. cruzi* proteasome β2 and β3 subunits superimposed with β4 and β5 subunits. The β2 (light blue) and β3 (dark blue) subunits were mapped unto the *L. tarentolae* corresponding proteasome subunits in PDB structure 6QM8. GNF6702 (dark shadow) binds with high affinity with subunit β3. The β4 (light orange) and β5 (dark orange) subunits were superposed to the β2 and β3 subunits, respectively. The β4 F24 and I29 residues, which are believed to play a role in GNF6702 sensitivity, are shown as sticks.

In the case of GNF6702, it is described to target the cell proteasome, specifically an allosteric site in the proteasome β4 subunit in close proximity to the catalytic site of the β5 subunit (Khare et al., 2016). A protein BLAST search in TriTryp found that the sequence corresponding to the proteasome β4 subunit described by Khare and co-workers is annotated as the β2 subunit of the *T. cruzi* CL Brener Esmeraldo-like strain (TcCLB.510287.30; UniProtKB ID Q4CU77), which was not found in the top 3% binders for GNF6702. Compared to the binding site described in the original article, which is situated adjacent to the residues F24 and I29 of the β4 subunit, near the β5 subunit, the pocket predicted by P2Rank and used in the docking was not found near those residues. Similar to the case of the FeSOD, the binding site might be formed in the junction of the two protein subunits, and so the correct binding site could not have been predicted. Nevertheless, it is noteworthy that position 1 out of 55 from GNF6702 top binders is occupied by the proteasome β3 subunit (TcCLB.506779.50, UniProtKB Q4DHA9). Mapping this subunit’s model onto the *Leishmania tarentolae* proteasome structure 6QM8, it can be visualized that the β3 subunit predicted binding site is in close proximity to the actual proteasome β2 subunit, which a BLAST search confirmed it to be the *T. cruzi* CL Brener Non-Esmeraldo-like (TcCLB.508461.430, UniProtKB Q4E4R6). A superposition of the β4 Q4CU77 subunit on the β2 Q4E4R6 subunit shows that the loop containing the F24 and I29 residues of β4 subunit matches the β2 loop in proximity to the predicted β3 binding site (**Figure 7**). A possible explanation would be that this loop plays an important role in GNF6702 sensitivity, and thus the proteasome β3 subunit could be a reasonable target for this ligand.

### Mitochondria-affecting compounds

In the case of compounds C16-2, C26-6 and C8-3, they are all proposed to act at the mitochondrial level. These three compounds appear to cause a bioenergetic collapse of the cell (2021a; 2021b; Martín-Escolano et al., 2018a). We found that the respiratory complex I NADH-ubiquinone oxidoreductase (TcCLB.506513.190, Q4DPI1), the catalytic first step in the electron transport chain, was selected in the top 3% in both C16-2 (position 35 out of 56) and C26-6 (position 2 out of 56), which would agree with what is described about these two compounds. Besides, C16-2 is a 3-alkoxy-1-vinylindazoles compound and thus belongs to the indazole family for which information about their mechanism of action on *T. cruzi* is scant in the literature. Some works suggest that indazoles are able to lead both the formation of ROS through their nitro group and also inhibit the trypanothione reductase (Aguilera-Venegas et al., 2013). Unfortunately, we did not find any of those enzymes in the top 3% reported for these ligands. Notably, dihydroorotate dehydrogenase (fumarate) (TcCLB.508375.50, Q4D3W2) was found in position 11 (out of 56) in C26-6 and 18 (out of 56) in C16-2, and aspartate carbamoyltransferase (TcCLB.507091.50, Q4DGV1) and orotidine-5-phosphate decarboxylase/orotate phosphoribosyltransferase (TcCLB.508373.29, Q4CSV7) were respectively found in positions 3 and 24 out of 56 in C16-2. These three enzymes are involved in the pyrimidine synthesis pathway, essential for parasite survival (Inaoka et al., 2017). Thus, it could be interesting to perform inhibition studies with these enzymes in search of a possible novel mechanism of action of indazoles compounds.

For C8-3, we retrieved the ATP synthase F_1_ subunit gamma (TcCLB.511145.60, Q4D1H0), mediator of the final step in the electron transport chain, selected in position 32 out of 56, which could also be the target of this compound. Interestingly, we also found trans-sialidase enzymes (TcCLB.505931.30 Q4CWF1, TcCLB.506515.29 Q4CPR9, TcCLB.508089.10 Q4CYW1 and TcCLB.509817.50 Q4CZP0) at positions 7, 8, 12 and 18 out of 56 for this same compound. C8-3 lightly resembles some benzoic acid derivatives that have been described to target the trans-sialidase protein family by another virtual screening study (Vázquez-Jiménez et al., 2021). Additionally, the presence of fluorine atoms, aside from increasing their metabolic stability and membrane permeation, could be involved in protein-ligand short contacts further increasing C8-3 binding affinity (Zhou et al., 2009).

### Glycosome-affecting compounds

Finally, C7-4 is a polyamine compound based on the well-known tripodal polyamine tris(2-amimoethyl)amine moiety (Martín-Escolano et al., 2019). Martin-Escolano and co-workers performed metabolism excretion, mitochondrial membrane potential and SOD-inhibition studies in order to decipher C7-4 mechanism of action. Their results showed that C7-4 anti-*T. cruzi* activity could be related to its effect at the glycosomal level (Martín-Escolano et al., 2019). In our analysis, we found a TcCLB.507009.10 (Q4DC12) glycosomal membrane protein, also annotated as Gim5A protein, occupying position 24 out of 55 in the top selected binders. It has been described that this protein might play an important role in the parasite transition from proliferative to stationary phase (Avila et al., 2018). In *T. brucei*, Gim5A and Gim5B are the most abundant glycosome membrane proteins, and depletion of the latter is lethal for the bloodstream form (Maier et al., 2001). However, there is no evidence of this protein in *T. cruzi*. On the other hand, polyamines are polycationic compounds essential for the growth and function of *T. cruzi* parasites, including cellular processes like the synthesis of trypanothione (Talevi et al., 2019). The thiol-polyamine metabolism of *T. cruzi* has been previously shown to be a suitable drug target due to its unique configuration and dependency on external supply (Reigada et al., 2018; Talevi et al., 2019). We found that C7-4 targeted spermidine synthases, the enzyme that converts putrescine into spermidine, in positions 43 (TcCLB.503855.20, Q4DBH6) and 48 (TcCLB.504033.130, Q4DR69) out of 55 in the top 3%. In addition, trypanothione reductase (TcCLB.504507.5, Q4CMQ7) was found at position 53. Our docking results showed that C7-4 is allocated in the flavin adenine dinucleotide (FAD) coenzyme site of the trypanothione reductase (Garrard et al., 2000; Beltran-Hortelano et al., 2017). Specifically, the fluorene moiety of C7-4 accommodates at the catalytic site near Cys53 and Cys58 residues, similarly to other polyamine derivatives with large substituents (Garrard et al., 2000). Altogether, the previously performed *in vitro* assays and our docking results could suggest multiple targeting for this compound.

## Conclusions

AlphaFold models of *T. cruzi* proteins open the way to new opportunities in drug discovery against this parasite, allowing to explore targets that have lacked structural information to date. With the aim to validate the application of this resource for computational drug screening purposes, we selected compounds with known targets or effects and launched an inverse virtual screening against the AlphaFold *T. cruzi* proteome. We found that some of the targets derived from the computational analysis successfully matched their experimentally described targets, while others showed a more nuanced result. The work performed identified some caveats of the virtual approach that must be taken into consideration. For instance, the quality of the models had a great variability between proteins, and given that precise residue positions and orientations are paramount in virtual drug screening experiments, we had to discard many of the structures from the subsequent docking simulations. Recently, a new pipeline based on AlphaFold has been developed, focused on improving the quality of models for trypanosomatids (Wheeler, 2021). This would provide very useful for “classical” virtual screening experiments, where usually just a few protein targets are studied, and so new models could be generated for these. Since models available at the AlphaFold Protein Structure Database only consider monomeric proteins, they cannot illustrate multimeric complexes and thus the binding sites formed in the interactions between subunits. This issue might be circumvented in the near future, as a new AlphaFold multimeric algorithm has been developed. On the other hand, some fine-tuning will be necessary for protein models that have post-translational modifications such as propeptides that need to be cleaved, or those proteins that have cofactors in their binding sites which must be considered to correctly predict binding affinities with ligands. Additional steps could be taken in order to further pinpoint suitable targets for a compound. For example, excluding from the top binders those proteins that are non-essential or those with multiple gene copies, and also prioritizing proteins which exert a high flux control on specific metabolic pathways (Olin-Sandoval et al., 2012). Furthermore, performing this inverse virtual screening pipeline with non-active analogs of the studied compound would also help to validate their specificity to any particular target; however, this is only doable with specific compound families and not in large-scale screenings such as the one we present here. All these steps, together with the results of phenotypic screening experiments, would help to propose a list of targets to be finally tested in an experimental setting. Despite these limitations, AlphaFold appears to be an extremely useful tool to study the 3D-space location of *Trypanosoma cruzi* proteins. Crystal structures deposited in PDB will be the gold standard, but these are scarce for neglected parasites, for which AlphaFold can contribute to fill (part of) the gap. While caution is advisable when using these models, some of them can show a high degree of quality, even comparable to PDB structures. Thus, they could be used not only for target deconvolution, but also for virtual screenings of chemical entities from diverse origin and nature in the search of new drugs to treat Chagas disease.

## Data Availability Statement

The original contributions presented in the study are included in the article/**Supplementary Material**. Further inquiries can be directed to the corresponding authors.

## Author Contributions

AR-L: conceptualization, methodology, software, formal analysis, investigation, writing - original draft, and visualization. NM-P: conceptualization, methodology, investigation, and writing - original draft. JB: validation and writing - review and editing. JG: validation and writing - review and editing. JA: conceptualization, validation, writing - review and editing, and supervision. All authors contributed to the article and approved the submitted version.

## Conflict of Interest

The authors declare that the research was conducted in the absence of any commercial or financial relationships that could be construed as a potential conflict of interest.

## Publisher’s Note

All claims expressed in this article are solely those of the authors and do not necessarily represent those of their affiliated organizations, or those of the publisher, the editors and the reviewers. Any product that may be evaluated in this article, or claim that may be made by its manufacturer, is not guaranteed or endorsed by the publisher.
