# Supplementary material for: The Use of AlphaFold for In Silico Exploration of Drug Targets in the Parasite Trypanosoma cruzi
Source: Front Cell Infect Microbiol. 2022 Jul 14;12:944748. doi: 10.3389/fcimb.2022.944748 (PMC9329570; doi:10.3389/fcimb.2022.944748)
Supplement: Supplementary file 1 [file Table_1.docx]

| **Drug** | **Reference (Ref. ID in ClinicalTrials.gov)** |
| --- | --- |
| Benznidazol | NCT00123916 |
| Nifurtimox | NCT04274101 |
| Fexinidazole | NCT02498782 |
| Posaconazole | NCT01162967 |
| E1224/Ravuconazole | NCT01489228 |
| Amiodarone | NCT03193749 |

**Table S1.** Selected drugs with anti-*T. cruzi* activity in clinical trials.
