# Supplementary material for: The Use of AlphaFold for In Silico Exploration of Drug Targets in the Parasite Trypanosoma cruzi
Source: Front Cell Infect Microbiol. 2022 Jul 14;12:944748. doi: 10.3389/fcimb.2022.944748 (PMC9329570; doi:10.3389/fcimb.2022.944748)
Supplement: Supplementary file 2 [file Table_2.docx]

**Table S2.** Selected drugs that inhibited *T. cruzi* equally or superior to the reference drug BNZ in a chronic model of Chagas disease.

| **Drug/Compound** | ***In vitro* activity** | ***In vivo* activity** | **Reference** |
| --- | --- | --- | --- |
| **GNF6702** | IC_50_ = 0.120 ± 0.0026 µM (Tulahuen β-gal)  TC_50_ ˃ 50 µM (macrophagues) | GNF6702 dosed twice-daily at 10mg/kg matched the efficacy of benznidazole at 100 mg/kg once-daily; all but one treated mouse had no detectable parasites by qPCR in blood, colon or heart tissue, even after 4 weeks of immunosuppression. | (Khare et al., 2016) |
| **Hydroxymethyl- nitrofurazone (NFOH)** | IC_50_ = 3.7 µM  (CL Luc-Neon) | Mice treated with NFOH (at 100 mg kg^−1^ for five consecutive days) reduced parasitaemia to background levels. After immunosuppression, 50% (3/6) of chronically infected mice treated with NFOH were assessed as cured. | (Scarim et al, 2021) |
| **Clofazimine** | At 5 µM:   - Infected cells < 5% - No amastigotes/cell < 2 (reduction > 3-fold than control) (Y-GFP) | Clofazimine (at 30 mg/kg for 30 days) was superior to BNZ (at 100mg/kg) at reducing parasite levels in cardiac muscles. Clofazimine treated group showed a quantitative and qualitative reduction in parameters of inflammation compared with untreated mice. | (Sbaraglini et al., 2016) |
| **Benidipine** | At 2.5 µM: No amastigotes/cell < 5 (reduction > 3-fold than control) (Y-GFP) | Benidipine (at 15 mg/kg for 30 days) was superior to BNZ (at 100 mg/kg) at reducing parasite levels in cardiac muscles and skeletal muscle. | (Sbaraglini et al., 2016) |
| **C9-1** | IC_50_ = 3.6 ± 0.3 µM (Arequipa); IC_50_ = 5.8 ± 0.5 µM (SN3); IC_50_ = 3.8 ± 0.3 µM (Tulahuen)  TC_50_ = 300 ± 24.1 µM (Vero cells) | C9-1 (at 20 mg/kg for 5 consecutive days) showed lower parasitaemia and higher curative rates than BNZ (same dose), also with lower toxicity. After immunosuppression, C9-1-treated mice showed a reactivation of parasitaemia of 12% in acute and 20% in chronic phases (BNZ 75% and 51%, respectively). It showed 89 % and 78 % of parasite-free organs/tissues in the acute and chronic phases (BNZ 33 % and 55 %, respectively). | (Martín-Escolano et al., 2018b) |
|  | | | |
| **Drug/Compound** | ***In vitro* activity** | ***In vivo* activity** | **Reference** |
| **C16-2** | IC_50_ = 7.4 ± 04 µM (Arequipa); IC_50_ = 5.8 ± 06 µM (SN3); IC_50_ = 12.4 ± 1.3 µM (Tulahuen)  TC_50_ = 266.5 ± 10.7 µM (Vero cells) | C-16-2 (at 20 mg/kg for 5 consecutive days exhibited better in vivo trypanocidal activity than BZN (same dose). In acute phase it showed a similar activity profile to BNZ. After immunosuppression, C16-2-treated mice showed a reactivation of parasitaemia of 30% and 35% in acute and chronic phases (BNZ, 75% and 51%, respectively). It showed 67% parasite-free organs/tissues for both phases (BNZ 33 and 55% for acute and chronic phases, respectively). | (Martín-Escolano et al., 2018a) |
| **C8-3** | IC_50_ = 0.8 ± 02 µM (Tulahuen)  TC_50_ = 44.3 ± 2.7 µM (Vero cells) | C8-3 (at 20 mg/kg for 5 consecutive days) showed higher efficacy than BZN (same dose). In acute phase it showed a similar activity profile to BNZ. After immunosuppression, C8-3-treated mice had reactivations of 67% in acute and 44% in chronic phases (BNZ 81% and 68%, respectively). C8-3-treated mice showed 3/7 and 4/7 parasite-free sites in acute and chronic phases (BNZ 2/7 and 3/7; respectively). | (Martín-Escolano et al., 2021b) |
| **C7-4** | IC_50_ = 4.8 ± 0.4 µM (Arequipa); IC_50_ = 19.7 ± 2.7 µM (SN3); IC_50_ = 15.1 ± 1.3 µM (Tulahuen)  TC_50_ = 1765.4 ± 158.4 µM (Vero cells) | C7-4 (at 20 mg/kg for 5 consecutive days) has a broader spectrum of action, improved efficacy and lower toxicity than BNZ (same dose). In the acute phase, C7-4 caused a reduction of the parasitaemia similar to BZN. After immunosuppression, C7-4-treated mice showed a parasitaemia reactivation of 16.3% in acute and 5.9% in chronic phases (BNZ 75% and 51.2%, respectively). C7-4-treated mice showed 2/9 and 1/9 tissues infected in the acute and chronic phase (BNZ 6/9 and 4/9, respectively). | (Martín-Escolano et al., 2019) |
|  | | | |
| **Drug/Compound** | ***In vitro* activity** | ***In vivo* activity** | **Reference** |
| **C9-5** | IC_50_ = 6.2 ± 0.3 µM (Arequipa); IC_50_ =6.6 ± 0.6 µM (SN3); IC_50_ =3.9 ± 0.4 µM (Tulahuen)  TC_50_ = 106.9 ± 11.0 µM (Vero cells) | C9-5 (at 20 mg/kg for 5 consecutive days) had better antichagasic activity than BZN (same dose). In acute phase, it decreases the parasitaemia peak similarly to BNZ. After immunosuppression, C9-5-treated mice showed a reactivation of 25% and 20% in the acute and chronic phases (BNZ 65% and 40%, respectively). C9-5 showed 50% (acute phase) and 63% (chronic phase) of organs/tissues free of parasites in acute and chronic phases (BNZ 25% and 38%, respectively). | (Paucar et al., 2019) |
| **C26-6** | IC_50_ = 5.4 ± 0.7 µM (Arequipa); IC_50_ =3.9 ± 0.5 µM (SN3); IC_50_ = 4.9 ± 0.3 µM (Tulahuen)  TC_50_ = 133.9 ± 11.3 µM (Vero cells) | C26-6 (at 20 mg/kg for 5 consecutive days) demonstrated a better activity profile that BZN (same dose). It showed low parasitaemia levels over the acute phase. After immunosuppression, C26-6 showed a reactivation of 65 and 60% in acute and chronic phases respectively, being slightly more effective than BZN. Regarding parasites on organ, C26-6 showed a better profile than BNZ, 42.9% of parasite-free organs/tissues in both phases. | (Martín-Escolano et al., 2021a) |

The IC_50_ values shown are against *T. cruzi* amastigote forms.
